# Effectiveness and Safety of Trabeculectomy along with Amniotic Membrane Transplantation on Glaucoma: A Systematic Review

**DOI:** 10.1155/2020/3949735

**Published:** 2020-10-09

**Authors:** Tian-yi Shen, Wei-nan Hu, Wen-ting Cai, Hui-zi Jin, Dong-hui Yu, Jing-hui Sun, Jing Yu

**Affiliations:** ^1^Department of Ophthalmology, Shanghai Tenth People's Hospital, Tongji University, School of Medicine, Shanghai 200072, China; ^2^Ninghai First Hospital, Ninghai, Zhejiang 315600, China; ^3^Anhui University of Science and Technology, Huainan, Anhui 232000, China

## Abstract

**Purpose:**

To determine the effectiveness and safety of trabeculectomy along with amniotic membrane transplantation (AMT) for glaucoma.

**Methods:**

This systematic review was performed using RevMan 5.3. We searched PubMed, EMBASE, and the Cochrane Library and included studies published until September 2019. The treatment group included patients with AMT and trabeculectomy (group A), and the control group had only trabeculectomy (group B). We only included randomized controlled trials. The outcomes were intraocular pressure (IOP), complete success rate, number of antiglaucoma medications, and complications.

**Results:**

Five studies, including 174 eyes (87 eyes in the AMT group and 87 eyes in the control group), were eligible in this review. The parameters had no significant difference in heterogeneity between the AMT and control groups preoperatively. In the AMT group, the mean IOP was significantly lower at 3 and 12 months after operation (*P* < 0.0001 and *P* = 0.02, respectively), while the number of complete successes in the AMT group was significantly higher at 6 and 12 months (*P* = 0.02 and *P* = 0.003, respectively) compared with the control group. Complications, including a flat anterior chamber and hyphema, appeared to be decreased in the AMT group compared to the control group (*P* = 0.02 and *P* = 0.02, respectively). No differences were observed in the number of antiglaucoma medications, hypotony, encapsulated bleb, or choroidal detachment.

**Conclusion:**

Compared with only trabeculectomy, it is more efficient and safer to add AMT to trabeculectomy during glaucoma filtering surgery.

## 1. Introduction

Glaucoma is a group of progressive optic neuropathies characterized by degeneration of retinal ganglion cells and their axons, resulting in cupping, a distinct appearance of the optic disc, and irreversible visual loss [1, 2]. The number of people with open-angle glaucoma (OAG) and angle-closure glaucoma (ACG) will increase to 79.6 million by 2020 worldwide, making it the second leading cause of blindness in the world [3].

The main treatment for glaucoma aims to reduce intraocular pressure (IOP) to slow down the process of vision loss. Medications and laser or incisional surgeries are conventional methods for reducing IOP. Trabeculectomy is the most common incisional surgical procedure, commonly performed in patients with medically uncontrolled glaucoma [4, 5]. However, postoperative fibrosis that most commonly occurs at the episclera leading to bleb failure months or years after filtering glaucoma surgery has limited the success rate of the treatment [6, 7]. For fear of these complications, the use of antifibrotic drugs, such as mitomycin-C and 5-fluorouracil, remains the standard for augmented trabeculectomy [8, 9]. Nonetheless, in some cases, it does not achieve a good filtering effect despite the use of the antifibrotic agents. Besides, endophthalmitis and hypotony may occur [10].

The amniotic membrane (AM) is the innermost layer of the fetal membranes. It is considered to be immunologically inert and possesses several physiologic properties, including inhibition of scarring, inflammation, angiogenesis, and provides a substrate for epithelial cell growth and attachment [11]. It is used as a biological tool due to its special structure, biological properties, and immunologic characteristics, which have already been applied in the treatment of burn lesions and for surgical wound covering to avoid collusion [12]. Amniotic membrane transplantation (AMT) has also been widely used in ophthalmic surgery to provide an alternative for corneal and conjunctival reconstruction, including limbal stem cell deficiency, ocular burn, pterygium, tumors, and symblepharon [13–17]. Zhang et al. found that the use of amniotic membranes along with trabeculectomy in primary congenital glaucoma can be an effective surgical method [18].

However, there is a lack of consensus regarding the effectiveness and safety of trabeculectomy with AMT compared with trabeculectomy alone in glaucoma. The objective of this systematic review was to analyze the IOPs and success rate along with complications after these two types of surgery.

## 2. Materials and Methods

### 2.1. Search Strategy

PubMed, EMBASE, and Cochrane Central Register electronic databases were searched from inception to March 2020, using the key words “glaucoma,” “amniotic membrane transplantation,” and “trabeculectomy.” The search was limited to English language reports without publishing time restriction.

### 2.2. Inclusion and Exclusion Criteria

Qualified studies included in the study must meet the following criteria: randomized controlled trial (RCT) design without restriction to language or type of publication, participants of all ages with medically uncontrolled glaucoma, use of AMT along with trabeculectomy as a treatment, a trabeculectomy treatment control group, and at least one year of follow-up. Both preoperative and postoperative outcome measures were obtained in each article. Accordingly, variables such as gender, type of glaucoma, and type of amniotic membrane were all potential sources of heterogeneity. Exclusion criteria were as follows: reviews, meetings, letters, animal studies, case reports, non-RCT design, studies without comprehensive data, use of different evaluation outcomes, and some duplicates.

### 2.3. Data Extraction and Quality Assessment

TY Shen and WN Hu independently filtered the titles and abstracts to remove obviously irrelevant reports. TY Shen, WN Hu, and WT Cai examined full-text reports and abstracts to determine the compliance with inclusion criteria. Agreements of detailed evaluation were reached after discussion by three reviewers. The studies which report the similar clinical trials were excluded and reserved one after extraction.

The guidelines in the Cochrane Handbook for Systematic Reviews of Interventions (version 5.1.0, Oxford, UK) [19] were adopted to validate the quality of all included articles. Data, such as the first author, year, country, randomized counts, age, sex, study design, type of surgery, duration of follow-up, clinical outcomes, and complications, were analyzed. The main clinical outcome was IOP. Secondary outcomes, including complete success rate, number of antiglaucoma medications, and complications, were extracted. Additionally, 12 months was set as the shortest follow-up period.

### 2.4. Statistical Analysis

RevMan 5.3 was performed for both fixed-effects and random-effects models in this review. To compare outcomes of different groups, odds ratios (ORs) and 95% confidence intervals (CIs) were used. *P* values were considered to be statistically significant at less than 0.05 level. Heterogeneity was evaluated with an *I*^2^ test in which more than 50% was considered to be significant. If significant heterogeneity was observed (*P* ≤ 0.05), a random-effects model was used for analyzing the data; otherwise, a fixed-effects model was performed (*P* > 0.05). Forest plot was estimated to show the comparison clearly [20, 21].

## 3. Results

### 3.1. Literature Search

**Figure 1:**
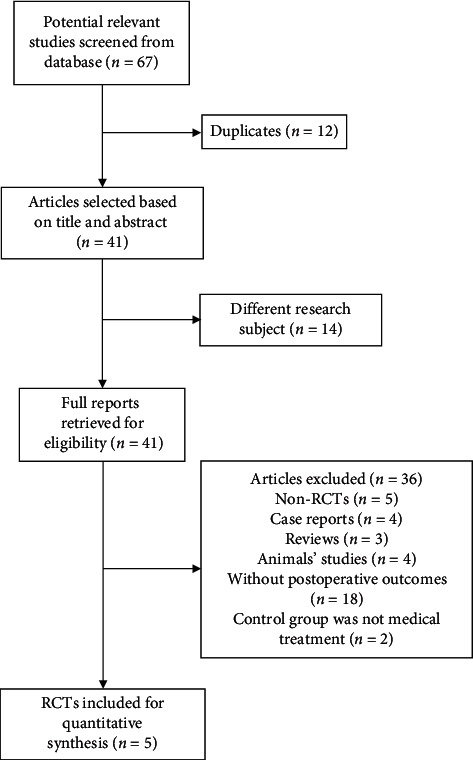
Flowchart of search strategy in this systematic review. RCT, randomized controlled clinical trial.

A total of 67 articles were identified by the original literature search (Figure 1), including 12 duplications. Reviewing the titles and abstracts, 41 articles were identified to be eligible for the full text of articles. Furthermore, 36 articles were excluded for the following reasons: non-RCTs, 5; case reports, 4; reviews, 3; animal studies, 4; without detailed outcomes, 18; and control group did not have trabeculectomy treatment, 2. As a result, 5 papers were available. Figure 1 is the flowchart of the literature retrieval progress.

### 3.2. Description

Table 1 shows the characteristics of the five trials. All of the studies included were RCTs. Totally, 174 eyes of 151 participants were identified with the size of the population ranging from 30 to 40, comprising 85 (56.3%) males and 66 (43.7%) females. The control group contained 87 patients as did the study group. Participants included all ages. The follow-up period ranged from 12 to 24 months, the minimum being 12 months. We used blinded fashion to assess the studies. Studies from Egypt, Germany, India, Brazil, and China were included.

### 3.3. Quality Assessment

**Figure 2:**
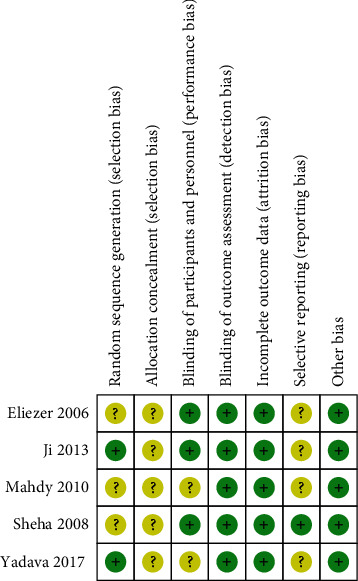
Methodological quality summary: authors' judgments about each methodological quality item for each included study. Note: + represents yes; ? represents unclear.

We used the bias assessment tool, which was recommended by the Cochrane Collaboration for RCTs.  Figure 2 shows the quality of the included studies. Though all the eligible studies indicated the use of randomized controlled trials, only two of them clearly listed the random block permutation method. Sensitivity analysis showed no statistically significant differences by removing individual trial. Funnel plots were not used to assess publication bias as fewer than ten trials were available.

### 3.4. Primary Outcome

#### 3.4.1. Mean IOPs

**Figure 3:**
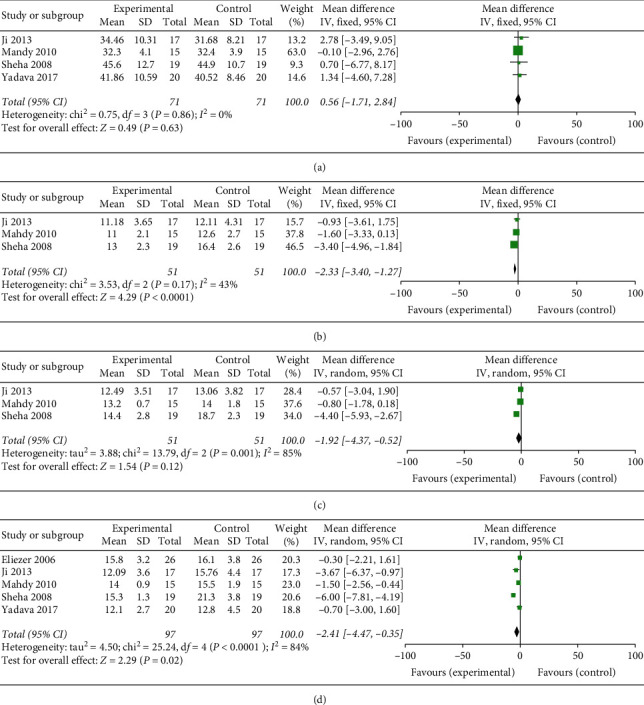
Forest plot comparison of IOP after treatment in the AMT and control group (a) preoperatively, (b) three months postoperatively, (c) six months postoperatively, and (d) one year postoperatively.

As shown in Figure 3, the mean IOPs decreased in both the trabeculectomy with AMT group (group A) and the trabeculectomy-alone group (group B). Before surgery, no difference appeared between two groups (WMD: 0.56, 95% CI: −1.71 to 2.84, and *P* = 0.63) (Figure 3(a)). At three months (WMD: −2.33, 95% CI: −3.40 to −1.27, and *P* ≤ 0.0001) (Figure 3(b)) and twelve months (WMD: −2.41, 95% CI: −4.47 to −0.35, and *P* = 0.02) (Figure 3(d)) after the treatment, the level of the mean IOP in group A was significantly lower than that in group B, while the mean IOP showed no difference at six months (WMD: −1.92, 95% CI: −4.37 to 0.52, and *P* = 0.12) (Figure 3(c)).

### 3.5. Secondary Outcomes

#### 3.5.1. The Number of Eyes with Complete Success

**Figure 4:**
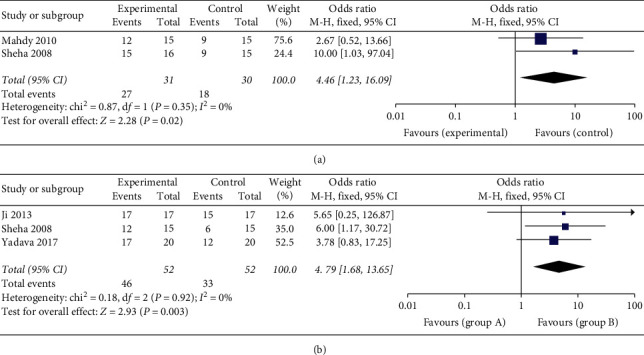
Forest plot comparison of the number of eyes with complete success after treatment in AMT and control group. (a) Six months postoperatively; (b) One year postoperatively.

There were four studies reporting the number of completely successful eyes after surgery at different time points. The number of successes shows significant improvement at six (OR: 4.46, 95% CI: 1.23 to 16.09, and *P* = 0.02) and twelve months (OR: 4.79, 95% CI: 1.68 to 13.65, and *P* = 0.003) after operation (Figure 4).

#### 3.5.2. The Number of Antiglaucoma Medications

**Figure 5:**
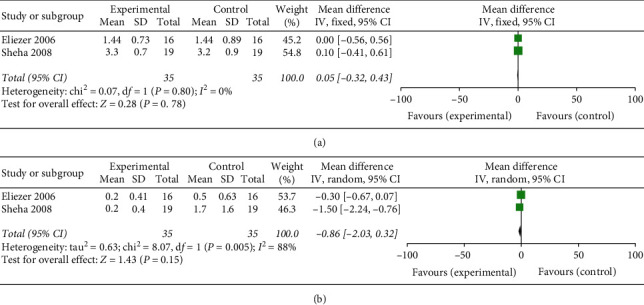
Forest plot comparison of the number of antiglaucoma medications after treatment in the AMT and control group (a) preoperatively and (b) one year postoperatively.

Two articles included the number of antiglaucoma medications before and after surgery. The difference between the two groups was not significant both before (WMD: 0.05, 95% CI: −0.32 to 0.43, and *P* = 0.78) and after the surgery (WMD: −0.86, 95% CI: −2.03 to 0.32, and *P* = 0.15) (Figure 5).

#### 3.5.3. Complications

**Figure 6:**
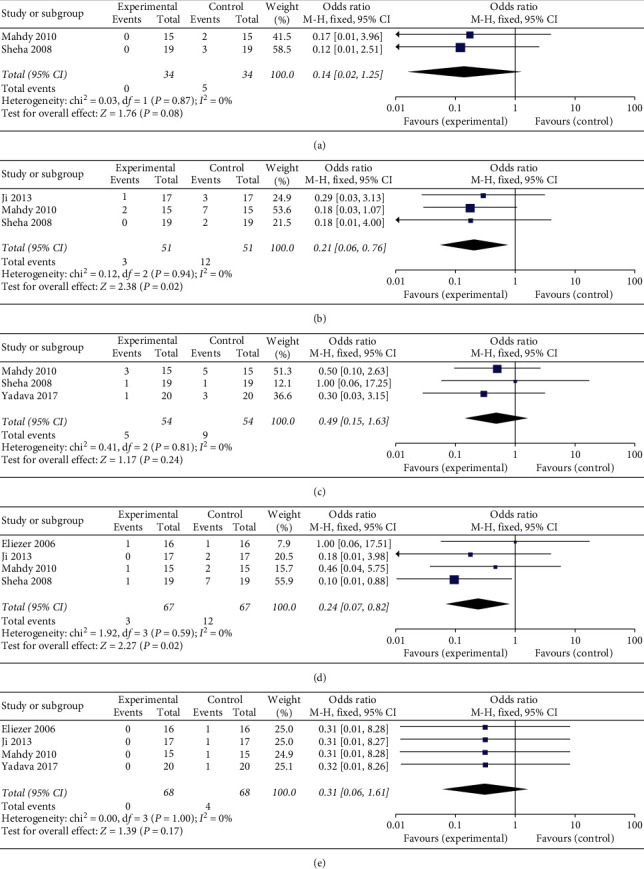
Forest plot comparison of complications after treatment in the AMT and control group. (a) Hypotony. (b) Flat anterior chamber. (c) Hyphema. (d) Encapsulated bleb. (e) Choroidal detachment.

The complication rates of hypotony, flat anterior chamber, hyphema, encapsulated bleb, and choroidal detachment were assessed after operation. A flat anterior chamber (OR: 0.24, 95% CI: 0.07 to 0.82, and *P* = 0.02) (Figure 6(b)) and hyphema (OR: 0.21, 95% CI: 0.06 to 0.76, and *P* = 0.02) (Figure 6(c)) appeared significantly decreased in group A compared with group B, while hypotony (OR: 0.31, 95% CI: 0.06 to 1.61, *P* = 0.17) (Figure 6(a)), encapsulated bleb (OR: 0.49, 95% CI: 0.15 to 1.63, *P* = 0.24) (Figure 6(d)), and choroidal detachment (OR: 0.14, 95% CI: 0.02 to 1.25, *P* = 0.08) (Figure 6(e)) showed no difference.

### 3.6. Heterogeneity

Heterogeneity appeared in some of the outcomes. This systematic review included all ages with glaucoma, of which Mahdy et al. [23] assessed the children with glaucoma. After excluding primary pediatric glaucoma, the analysis showed similar results as previous. The outcome indicated that our conclusions remained stable.

## 4. Discussion

Trabeculectomy has been widely used as the traditional filtering surgery to control IOP levels with antiglaucoma medications alone in glaucoma. However, the complications of fibrosis, which may affect visual function, made a permanent result difficult.

Due to the limitations mentioned above, AM became an adjuvant treatment. It has been shown to inhibit squamous metaplasia of the conjunctival epithelium, suppress inflammation and neovascularization, promote limbal stem cell expansion, and accelerate the corneal epithelium [27–29]. The AMT pathway may downregulate transforming growth factor-*β* signaling in cultured normal conjunctival and pterygium fibroblasts [30]. Moreover, amniotic membrane proteins can modulate the gene involved in apoptosis and reduce oxidative stress along with inflammatory responses in a hypoxic condition [31].

As the main target of glaucoma, elevated IOP is associated with glaucomatous optic nerve damage and visual field loss. Kimball et al. [32] found that IOP elevation in elder mice caused clear abnormalities in the density and movement of mitochondria as well as axonal integrity. The stability of IOP seems to be essential. Shao et al. [33] has proven the effectiveness of adding AMT to trabeculectomy, resulting in a controlled IOP and a functional filtration bleb sustained in rabbit glaucoma models, which were the same as the statistics shown in this review. IOP levels decreased to normal after treatment in the two groups, while the decline in the AMT group was more notable and stable. We came to the conclusion that trabeculectomy with AMT shows superiority in reducing IOP levels compared to trabeculectomy alone. At the same time, it produces a complete success rate at a high standard. We excluded qualified successes (having an IOP of 21 mm Hg or less with or without antiglaucoma medications) so that more valuable statistics could be acquired. Although the number of antiglaucoma medications was equal before and after surgery, the complete success rate can indicate an advantage in the AMT group.

Complications are a long-term index that can be used to qualify surgeries. Five kinds of complications, including hypotony, flat anterior chamber, hyphema, encapsulated bleb, and choroidal detachment were analyzed. Hypotony, flat anterior chamber, and choroidal detachment had a low probability when AM was inserted under the scleral flap, and they halted rapid drainage of the aqueous humor from the trabeculectomy site effectively, [22] though only cases of a flat anterior chamber had a significant decrease in the AMT group. Hyphema, the accumulation of blood in the anterior chamber, [34] can be seen after ocular trauma, intraocular surgery, or spontaneously, which can cause complications such as secondary hemorrhage and glaucoma [35]. On the basis of review, the AMT group was able to avoid this complication, which may reduce the recurrence of glaucoma. Collagen-producing fibroblasts are always the reason for encapsulated blebs, which may elevate IOP, leading the eye to be uncomfortable from the effects of a localized dellen [36]. The AMT group had a lower prevalence of fibroblasts than the control group, though without significance.

In conclusion, although only prospective studies were included, some limitations should not be overlooked. First, not every article explained the randomized strategy explicitly and convincingly. Second, some factors, such as the size of glaucoma, treatment before operation, surgeon skills, and surgical methods, could have affected our results. Third, for lack of an abundant number of eligible cases, some statistics were unable to be acquired, and a limitation exists regarding visual ability and other vision aspects. Overall, this review suggested that AMT is an effective and safe treatment in combination with trabeculectomy in glaucoma for its stable IOP level, high success rate, and low incidence rate of complications.

## Figures and Tables

**Table 1:** Patient characteristics in the included trials.

Reference	Year	Location	Inclusion criteria	Intervention	Eyes	Age, year (mean ± SD)	Sex (M/F)	Follow-up time (mos)	Outcomes
Sheha et al. [22]	2008	Germany	Refractory glaucoma	Trabeculectomy	19	56.6 ± 6	11/7	12	Mean IOPs; success rate; mean number of antiglaucoma medications; complications
				Trabeculectomy with AMT	19	57.6 ± 6.3	13/6		
Mahdy et al. [23]	2010	Egypt	Primary pediatric glaucoma	Trabeculectomy	15	6 ± 2.1	7/6	18	Mean IOPs; success rate; complications
				Trabeculectomy with AMT	15	6 ± 1.8	5/7		
Yadava et al. [24]	2017	India	POAG; PACG	Trabeculectomy	20	54.65 ± 11.05	11/9	12	Mean IOPs; success rate; complications
				Trabeculectomy with AMT	20	50.95 ± 9.54	9/11		
Eliezer et al. [25]	2006	Brazil	POAG	Trabeculectomy	16	67.6 ± 8.0	11/5	12	Mean IOPs; BCVA; mean number of antiglaucoma medications; complications
				Trabeculectomy with AMT	16	68.3 ± 13.6	8/8		
Ji et al. [26]	2013	China	Chronic angle-closure glaucoma; POAG	Trabeculectomy	17	61.6 ± 13.4	10/7	24	Mean IOPs; success rate; complications
				Trabeculectomy with AMT	17				

POAG, primary open-angle glaucoma; PAGC: primary angle-closure glaucoma; AMT: amniotic membrane transplantation

## Data Availability

The data used to support the findings of this study are included within the article.
